# Lateral Release – Eine Verschlusstechnik für mittelgroße bis große Läsionen der Kopfhaut

**DOI:** 10.1111/ddg.70008

**Published:** 2026-08-04

**Authors:** Julian Kött, Daniel Czesla, Moritz Felcht

**Affiliations:** ^1^ Klinik und Poliklinik für Dermatologie und Venerologie Universitätsklinikum Hamburg‐Eppendorf, Hamburg; ^2^ Fleur Hiege Centrum für Hautkrebsforschung Universitätsklinikum Hamburg‐Eppendorf, Hamburg; ^3^ Zentrum für Dermatochirurgie St. Josefskrankenhaus Akademisches Lehrkrankenhaus der Medizinischen Fakultät Mannheim der Universität Heidelberg, y

## EINLEITUNG

Hauttumoren der Kopfhaut nehmen im Zuge der alternden Bevölkerung signifikant zu, wobei insbesondere das Basalzellkarzinom und das kutane Plattenepithelkarzinom dominieren.^1^, ^2^ Große Läsionen, die eine umfangreiche Tumorexzision erfordern, führen oft zu Defekten, welche die Möglichkeiten einer einfachen Rekonstruktionstechnik überschreiten. Neben der Größe der Läsionen stellt insbesondere die reduzierte Elastizität der Kopfhaut und deren Konvexität eine Herausforderung dar. Etablierte Verfahren sind Techniken wie Dehnungsplastik, Rotationslappenplastik, Rotationslappenplastiken von zwei Seiten, Transpositionslappenplastik, A‐T‐Lappenplastik, H‐Lappenplastik, sekundäre Wundheilung mit anschließender Spalthautdeckung, Teilverschluss und ergänzende Vollhauttransplantation oder freie vaskularisierte Lappenplastiken.^3^, ^4^, ^5^

Eine Möglichkeit die Spannung der Naht zu reduzieren sind Inzisionen der Galea neben dem Defekt, die es erlauben größere Defekte primär zu verschließen.^6^, ^7^ Russo beschreibt seine Erfahrung bei 119 Defektverschlüssen an der Kopfhaut mit Defektgrößen < 1 cm, 1–2 cm, 2–3 cm, 3–4 cm und > 4 cm.^7^ Eine lateral entfernte Inzision wurde in 22/33 der 1–2 cm großen Defekt genutzt. Zwei laterale Inzisionen wurden bei 14/21 Defekten mit 2–3 cm und drei laterale Inzisionen bei 7/14 der 3–4 cm großen Läsionen angewendet.^7^ Diese Technik möchten wir den JDDG‐Lesern vorstellen und unser operatives Vorgehen erläutern.

## TECHNIK

Der *Lateral Release* basiert auf einer zentralen Dehnungsplastik, ergänzt durch ein oder zwei seitliche Inzisionen. Die Inzision muss ausreichend tief erfolgen und die Galea aponeurotika miteinschließen, da diese die Mobilisierung am meisten behindert. Diese Technik ist aus unserer Sicht ein gutes Verfahren bei Kopfhautdefekten einer Größe von 3–4 cm.

**ABBILDUNG 1:**
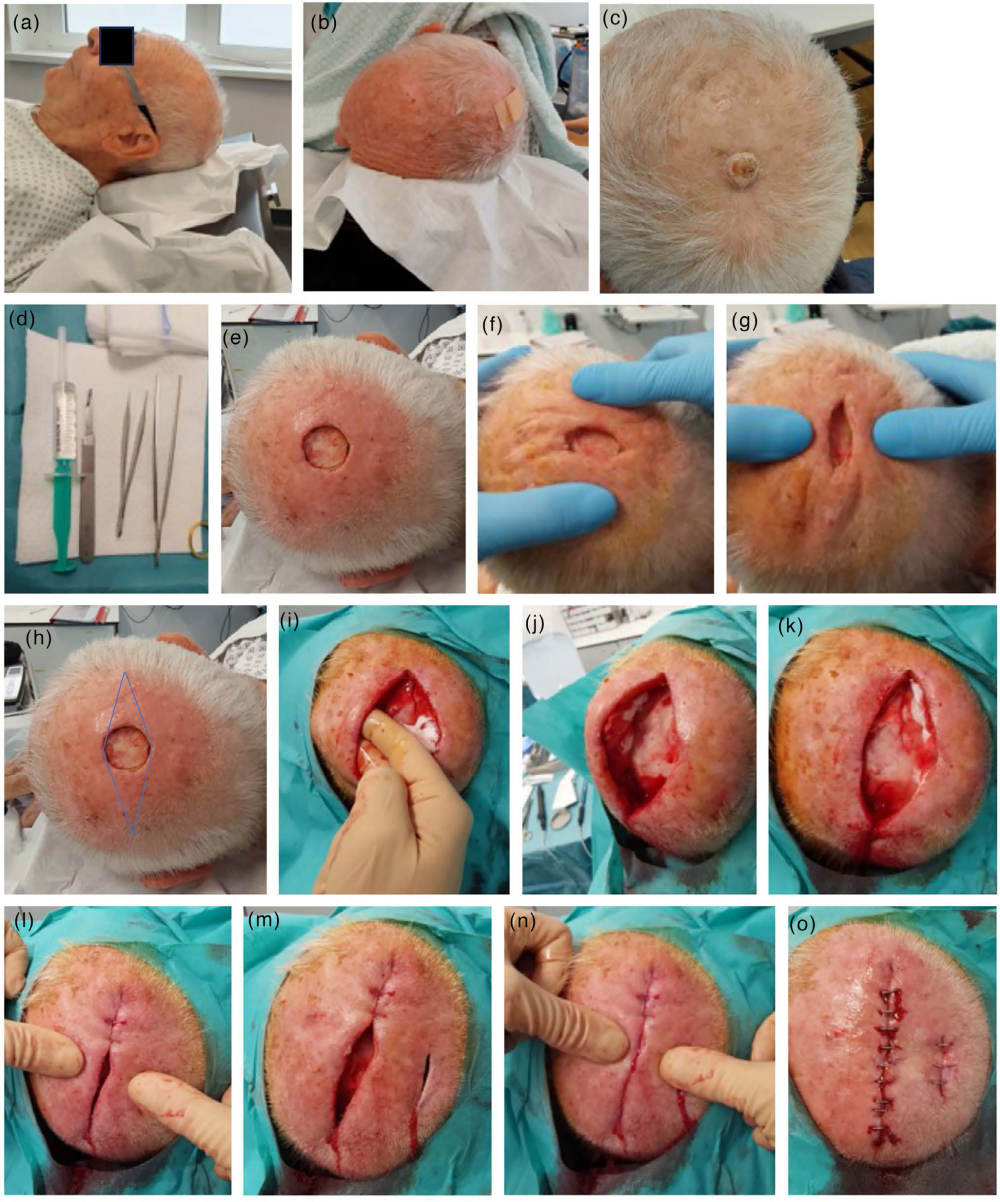
Detaillierte Darstellung der Lateral‐Release‐Technik: (ab) Der Patient wird horizontal gelagert; (c–e) Exzision eines Plattenepithelkarzinoms, vor der ersten Inzision erneute sterile Injektion von Lokalanästhetikum, Entstehung eines Defekts mit einem Durchmesser von 4 × 4 cm; (f, g) Der Kneiftest zeigt die größte Hautmobilität in kranialer und kaudaler Richtung; (h) Einzeichnen der Ausgleichsdreiecke zur Nutzung der größten Beweglichkeit; (i–k) stumpfe Präparation mit den Fingern und Einbringen steriler Kompressen in die präparierten Wundtaschen; (l) primärerWundverschluss, soweit spannungsfrei möglich, (m) Anlage eines Lateral‐Release‐Schnitts kaudal des Defekts; (n) nun ist ein spannungsfreier Verschluss möglich; (o) Vollständiger Verschluss mit Klammernähten.

1. *Lagerung*: Bei allen tiefen Eingriffen am Capillitium empfehlen wir eine horizontale Lagerung (ggf. auch in Seitenlagerung bei Befunden am Hinterkopf), um eine in der Literatur beschriebene Luftembolie in senkrechter Lagerung zu vermeiden (Abbildung 1a, b).^8^ Die Pathophysiologie für die Luftembolie ist bislang nicht vollständig verstanden und die Inzidenz sehr gering. Andere Operateure bevorzugen andere Lagerungen um die erhöhte Blutungsneigung bei horizontaler Lagerung zu vermeiden.
2. *Vollständige Exzision des Tumors* (Abbildung 1b–d). Dabei ist anzumerken, dass operative Eingriffe am Schädel trotz signifikanter Mengen an Lokalanästhesie als besonders schmerzhaft empfunden werden. Es ist anzunehmen, dass dies durch eine besonders gute Resorption des Lokalanästhetikums bedingt ist. Ein typischer Circulus vitiosus, den es zu vermeiden gilt, besteht darin, dass Schmerzen zu einer Blutdrucksteigerung führen, die wiederum eine verstärkte Blutung nach sich zieht. Dies erfordert eine intensivere elektrokaustische Hämostase, welche die Schmerzsymptomatik zusätzlich verstärkt. Aus diesem Grund ist es hilfreich vor dem ersten Schmerzreiz (gewöhnlich die Inzision) noch einmal steril Lokalanästhetikum zu injizieren (Abbildung 1d). Zur postoperativen Analgesie setzen wir routinemäßig Ibuprofen ein.
3. Es wird mit dem *Kneiftest* nach Pinkus überprüft, von welchen Seiten sich die Haut am besten mobilisieren lässt (Abbildung 1f, g).^9^
4. Wie gewöhnlich bei einer Dehnungsplastik werden *Ausgleichsdreiecke von 30°* gewählt, um die Spannung so weit wie möglich zu verteilen (Abbildung 1h, blaue Linien). Dies kann mit einem monopolaren Messer durchgeführt werden, um *(1)* möglichst zügig arbeiten zu können und den oben beschriebenen Circulus vitiosus für Schmerzen zu vermeiden und *(2)* direkt alle kleineren Gefäße des subdermalen Plexus zu veröden.
5. *Weite stumpfe Mobilisation im subgalealen Raum* (Abbildung 1I). Hier ist es essenziell, dass in der richtigen Schicht präpariert wird. Eine Präparation auf dem superfiziellen muskuloaponeurotischen System (SMAS) führt zu starken Blutungen und die dadurch notwendige Blutstillung resultiert teilweise in einer Schädigung des subdermalen Plexus, sodass sich Nekrosen bilden können. Man erkennt bei der Präparation, ob man in der richtigen Schicht ist, wenn *(1)* es wenig blutet und *(2)* ein stumpfes Lösen der Galea vom Periost, mit der Schere oder der Hand, ein charakteristisches knirschendes Geräusch erzeugt. Nach der Präparation kann es hilfreich sein, die neu entstandene Wundhöhle mit einer Kompresse auszutamponieren (Abbildung 1j, k). Dadurch soll die Haut vor dem Verschluss etwas vorgedehnt werden und der Wundrand ist besser für eine notwendige Blutstillung zugänglich.
6. Nach weiter Mobilisation zu beiden Seiten und guter Blutstillung erfolgt ein *Verschluss* mit versenkten hohen Koriumsnähten, soweit dies spannungsfrei möglich ist. Es verbleibt zentral ein Rest, der sich nicht verschließen lässt (Abbildung 1l).
7. Es erfolgt die Anlage eines oder ggf. auch zweier seitlich des Defektes gelegener tiefer Relaxationsinzisionen (Lateral Release*) parallel zu der Hauptachse* der Dehnungsplastik: Diese werden zentral angelegt und sind in der Regel nicht länger als die ursprüngliche Defektlänge (3–4 cm). Besser sind kürzere Schnitte (beispielsweise 80 % der Defektlänge) (Abbildung 1l). Wichtig ist, dass der Abstand zwischen dem Defekt und dem Lateral‐Release‐Schnitt ausreichend groß gewählt wird und er sollte nicht kürzer als die Länge des neu angelegten Zusatzschnittes sein. Zur Veranschaulichung: Wenn der Schnitt beispielsweise 3 cm lang ist, sollte der Abstand zwischen Lateral‐Release‐Schnitt und Defekt ebenfalls mindestens 3 cm betragen. Insbesondere sollte der Schnitt tief genug sein und die *Galea einschneiden*. Nur dadurch kommt die zusätzliche Beweglichkeit zustande. Gegebenenfalls ist noch eine weitere Mobilisation subgaleal über den/die *Lateral Release*‐Zugänge notwendig.
8. Jetzt lässt sich der zentrale Restanteil des *Primärdefektes spannungsfrei verschließen* (Abbildung 1n, o).
9. Danach erfolgt der *Verschluss des/der Lateral‐Release‐Zugangs/Zugänge* mit Koriumsnähten, Haut‐ oder Klammernähten (Abbildung 1o).

Durch den *Laterale Release* und die entsprechend laterale(n) Inzision(en) (ein oder zwei) wird die Fläche zur Unterminierung vergrößert, damit die Mobilität erweitert und so der Defektverschluss ermöglicht wird. Dies gelingt durch die Inzision der am wenigsten mobilen Schicht, der Galea aponeurotica.

Limitationen der Technik sind Größen von mehr als 5 cm, Fibrose oder Vernarbung durch Bestrahlung oder vorangegangene Operationen und damit einhergehende reduzierte Elastizität und Verschieblichkeit. Folgende Risiken müssen berücksichtigt werden: Im Allgemeinen Blutungen, Nachblutungen, Infektionen; zusätzlich abhängig von der Lokalisation okzipitale Neuropathie.

## DISKUSSION

**ABBILDUNG 2:**
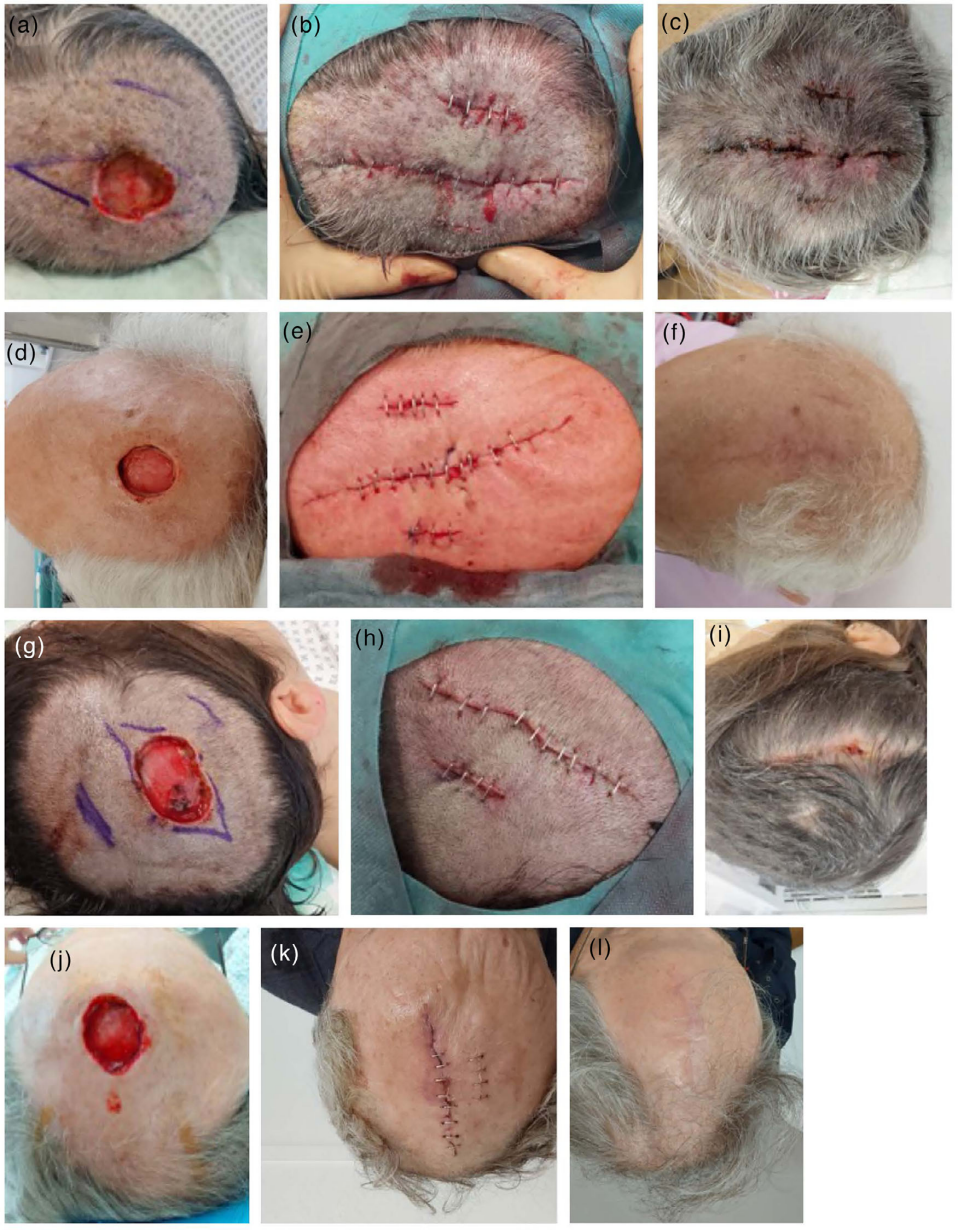
Klinische Beispiele der Lateral‐Release‐Technik (a) Kopfhautdefekt (2,8 × 3,8 cm) nach In‐sano‐Exzision eines Lentigo‐maligna‐Melanoms mit beidseitigem *Lateral Release* (b) Postoperatives Ergebnis nach Verschluss mittels Klammernaht (c) 2 Wochen postoperativ zeigen sich nach Entfernung der Klammern reizfreie Narbenverhältnisse (d) Kopfhautdefekt nach Exzision eines Lentigo‐maligna‐Melanoms (Tumordicke 0,4 mm; Defektgröße 4 × 4 cm) (e) Verschluss durch zwei Lateral‐Release‐Schnitte (f) Gutes postoperatives Ergebnis nach 3 Monaten (g) Kopfhautdefekt nach Exzision eines soliden Basalzellkarzinoms auf dem Boden eines Naevus sebaceus (Defektgröße 5 × 3 cm) (h) Verschluss durch eine Dehnungsplastik in Kombination mit einem *Lateral Release* (i) Guter postoperativer Befund nach 8 Wochen (j) Kopfhautdefekt nach Exzision eines ulzerierten Basalzellkarzinoms mit Nervenscheideninfiltration (Defektgröße 3 × 2,5 cm) (k) Verschluss unter Einschluss eines *Lateral Release* (l) Zufriedenstellendes postoperatives Ergebnis nach 6 Monaten.

**ABBILDUNG 3:**
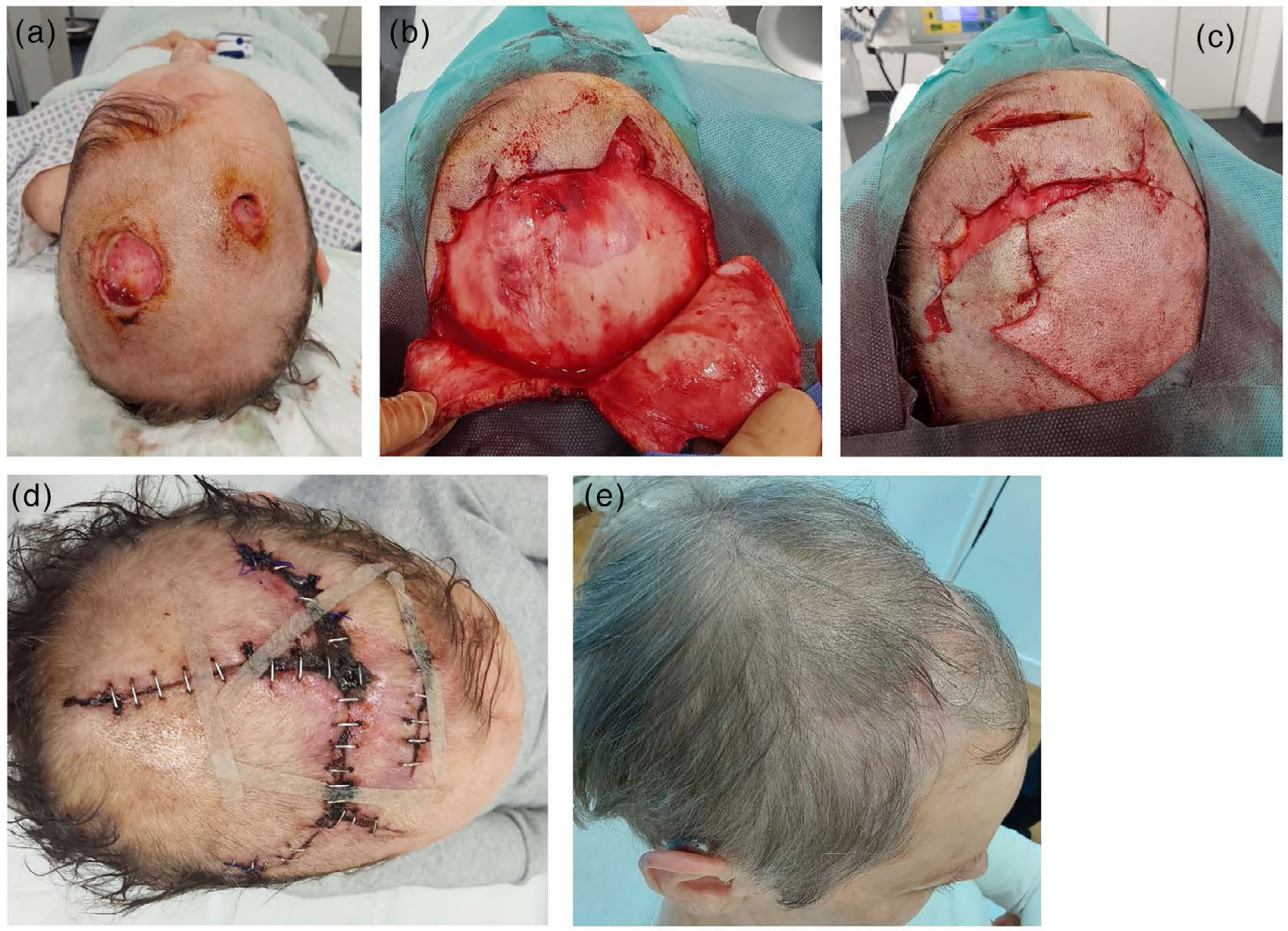
Klinisches Beispiel eines Lateral‐Release‐Schnitts bei zwei Rotationslappenplastiken (a) Kopfhaut mit zwei großen Defekten nach Rezidiv eines multilokulär infiltrativ wachsenden soliden Basalzellkarzinoms parietal links sowie eines multifokal wachsenden soliden Basalzellkarzinoms parietal rechts. (b–d) Der Verschluss erfolgte mittels zweier Rotationslappenplastiken, sechs Ausgleichsdreiecken, zwei Rückschnitten (back cuts) und einem Lateral Release an der Stirn. (e) Akzeptables postoperatives Ergebnis nach 5 Monaten.

Der *Lateral Release* stellt eine einfache und effektive Methode dar, um große Defekte der Kopfhaut nach Tumorexzision zu verschließen. Durch die gezielte Spannungsumverteilung wird ein primärer Wundverschluss ermöglicht, der in ästhetischer und funktioneller Hinsicht überzeugt (Abbildung 2). Diese Technik bietet sich an, da sie mit minimalem Material‐ und Zeitaufwand durchgeführt werden kann. Als Vorteile sind die reduzierte Spannung im zentralen Bereich der Wunde, kürzere Operationsdauer im Vergleich zu komplexen Rekonstruktionstechniken und ein gutes funktionelles und ästhetische Ergebnis zu nennen. Nachteilig bei Eingriffen am Capillitium sind insbesondere Schmerzen trotz hoher Dosen eines Lokalanästhetikums (siehe oben), mögliche Verletzungen von Venen an den Relaxationsschnitten sowie die seltene, aber schwerwiegende Komplikation einer Luftembolie. In der Regel wenden die Autoren den *Lateral Release* bei einer Dehnungsplastik an (Abbildung 2). Er kann aber auch hilfreich sein bei Kombinationen von Rotationslappenplastiken (Abbildung 3).

Baker et al. beschreiben für onkologische Defekte am Stamm und den Extremitäten den *modified bipedicled flap*, welcher ebenfalls einen seitlichen Schnitt zur Spannungsreduktion aufweist.^10^ Im Vergleich zum hier beschriebenen *Lateral Release* ist der *modified bipedicled flap* abgerundet und deutlich länger als der Schnitt am eigentlichen Defekt. Dadurch wird zwar die Spannung auf die Defektnaht verringert, jedoch die Gesamtdefektfläche deutlich vergrößert. Die laterale Inzision mit anschließendem Verschluss erinnert ebenfalls an den *Keystone flap*, der für gewöhnlich an den unteren Extremitäten zum Einsatz kommen kann.^11^

Im Vergleich zu Rotationslappenplastik, A‐T‐Lappenplastik, Rotationslappen von zwei Seiten, Transpositionslappenplastik, doppelte Rotationslappenplastik und H‐Lappenplastik benötigt der *Lateral Release* keine umfangreichen Hautverschiebungen oder zusätzliche Transplantate.^3^, ^5^ Im Vergleich zur sekundären Wundheilung mit gegebenenfalls folgender Spalthautdeckung erspart es eine nachfolgende Operation und minimiert das Infektionsrisiko durch den direkten Wundverschluss. Außerdem entfällt die teilweise recht lange Zeit der aufwendigen Wundpflege bei einer sekundären Wundheilung. Eine weitere operative Lösung wäre ein Teilverschluss in Kombination mit einer sekundären Wundheilung oder einer Spalthautdeckung des zentralen Defekts im weiteren Verlauf.^12^ Hier sind zahlreiche Verbandswechsel, die Infektionsgefahr bei offenem Defekt und ästhetisch die sichtbare Narbe als Nachteile zu nennen. Als vorteilhaft kann die Zeitersparnis intraoperativ angeführt werden. Darüber hinaus ist ein Teilverschluss in Kombination mit einer direkten Vollhauttransplantation möglich, jedoch ebenfalls ästhetisch mit einer bleibenden Narbe beziehungsweise Transplantat deutlich unterhalb des Hautniveaus und mit einem zusätzlichen Defekt an anderer Körperstelle verbunden, der ebenfalls gedeckt werden müsste.

### Schlussfolgerungen

Der *Lateral Release* bietet eine minimal‐invasive Lösung für den Verschluss großer Defekte der Kopfhaut. Die Technik ist leicht erlernbar, ressourcenschonend und bietet ein funktionell, ästhetisch und chirurgisch gutes Ergebnis.

## DANKSAGUNG

Open access funding enabled and organized by Projekt DEAL.

## INTERESSENKONFLIKT

Keiner.
